# Follow-up of transcatheter closure of congenital heart disease complicated with pulmonary arterial hypertension in children

**DOI:** 10.3389/fped.2025.1562782

**Published:** 2025-03-07

**Authors:** Yanyun Huang, Yuting Chen, Danyan Su, Suyuan Qin, Cheng Chen, Dongli Liu, Bingbing Ye, Yuqin Huang, Piaoliu Yuan, Yusheng Pang

**Affiliations:** ^1^ Department of Pediatrics, The First Affiliated Hospital of Guangxi Medical University, Nanning, Guangxi, China; ^2^ Difficult and Critical Illness Center, Pediatric Clinical Medical Research Center of Guangxi, The First Affiliated Hospital of Guangxi Medical University, Nanning, Guangxi, China

**Keywords:** transcatheter closure, pulmonary arterial hypertension, congenital heart disease, children, follow-up

## Abstract

**Background:**

Transcatheter closure is now the preferred treatment for congenital heart disease complicated with pulmonary arterial hypertension (CHD-PAH), but its long-term effects are not well understood. We aimed to assess the safety, effectiveness, and outcome of this procedure in children with CHD-PAH.

**Methods:**

We included 210 children with CHD-PAH at our hospital from 2012 to 2021 and collected their general, laboratory, echocardiographic, and hemodynamic data for analysis. A logistic regression analysis identified risk factors for persistent postclosure PAH (PP-PAH).

**Results:**

Among the 210 patients, 84.29% had mild PAH, 8.57% had moderate PAH, and 7.14% had severe PAH. The device was successfully implanted in 98.10% of patients. Early adverse events occurred in 12.14% (*n* = 25) of patients, with residual shunts and arrhythmia being the most common complications, each affecting 2.91% (*n* = 6) of patients. Most complications were minor and temporary, except for two cases of residual shunt—one required surgical repair, and one case of complete left bundle branch block led to occluder removal. Postintervention, pulmonary arterial pressure (PAP) decreased significantly, and cardiomegaly resolved. PP-PAH was detected in 13 patients (6.31%). Preoperative pulmonary arterial systolic pressure [odds ratio [OR] = 1.033, 95% confidence interval [CI] = 1.005–1.061, *P* = 0.019] and right ventricular diameter (OR = 1.111, 95% CI = 1.039–1.187, *P* = 0.002) were found to be risk factors for PP-PAH.

**Conclusion:**

Transcatheter closure is effective and safe for children with correctable CHD-PAH. Preoperative pulmonary arterial systolic pressure and right ventricular diameter are risk factors for PP-PAH.

## Introduction

Congenital heart disease (CHD), recognized as the most prevalent congenital anomaly, manifests in approximately 8 out of every 1,000 newborns (1). Despite advancements in diagnostic and therapeutic approaches, approximately 5%–10% of patients with CHD may develop pulmonary arterial hypertension (PAH) if the condition remains uncorrected, leading to significant morbidity and mortality (2). Persistent systemic-to-pulmonary shunting causes overflow in the pulmonary circulation, ultimately leading to irreversible remodeling of the pulmonary vasculature (3). Data suggest that PAH can be reversed by early closure of the congenital defect prior to the onset of vascular remodeling (4). Once pulmonary vascular remodeling becomes irreversible, defect closure is associated with accelerated disease progression (5). This renders decisions regarding the closure of the defect particularly challenging.

In current guidelines, pulmonary vascular resistance (PVR) and the ratio of pulmonary to systemic circulation (Qp/Qs) are considered the most critical reference indices for determining the indication for defect closure. The 2020 European Society of Cardiology (ESC) Guidelines suggest shunt closure for patients with PVR ≤ 5 wood units and Qp/Qs > 1.5 (6). The 2019 European Pediatric Pulmonary Vascular Disease Network (EPPVDN) recommends that a PVR index (PVRI) < 6 wood units/m^2^ is an indication for shunt closure, whereas a PVRI > 8 wood units/m^2^ is a contraindication (7). Unfortunately, these cutoff values rely largely on expert opinions and lack prospective data support. Despite a Qp/Qs > 1.5, some patients still develop or experience worsening PAH after defect correction. Consequently, decisions regarding shunt closure should be based on a comprehensive evaluation of all the clinical information, rather than relying solely on hemodynamic data. This conclusion highlights the need for follow-up and assessment of CHD-PAH patients after defect closure. Several reports have suggested that defect closure in patients with CHD-PAH may be linked to favorable outcomes (8–12). Conversely, other studies have indicated that PAH may continue to progress despite closure of the defect (13, 14). However, most prior studies involved patent ductus arteriosus (PDA) or atrial septal defect (ASD) patients and had relatively small sample sizes. Some of these studies were performed in adults. Little has been published focusing specifically on the outcomes of children with CHD-PAH after correction.

Percutaneous transcatheter closure of CHD-PAH is now the preferred treatment at many centers because of its minimal invasiveness, quick recovery, ability to monitor pulmonary arterial pressure (PAP), and the advatange of trial occlusion. However, little is known about its outcomes. In the present study, we retrospectively evaluated the safety, effectiveness, and outcomes of transcatheter closure in children with CHD-PAH.

## Methods

### Patients and definitions

The study was conducted at the First Affiliated Hospital of Guangxi Medical University, enrolling CHD-PAH patients who had transcatheter closures between January 2012 and December 2021. PAH is diagnosed when the mean pulmonary arterial pressure (mPAP) is >20 mmHg via cardiac catheterization (15). The patient inclusion criteria were as follows: (1) meeting the PAH diagnostic criteria; (2) having simple CHD, including PDA, ASD, ventricular septal defect (VSD), or their combination; (3) a PVRI < 6 wood units/m^2^; (4)age < 18 years; and (5) availability of complete and traceable medical records. Patients with other causes of PAH were excluded from the study. The patients were categorized into three groups according to the mPAP levels: mild (20–40 mmHg), moderate (41–55 mmHg), and severe (>55 mmHg). After the intervention, PAH was evaluated by transthoracic echocardiography (TTE) during follow-up. PAH was identified by a pulmonary arterial systolic pressure (PASP) exceeding 35 mmHg and categorized as mild (35–50 mmHg), moderate (50–70 mmHg), or severe (>70 mmHg) (16–18). Persistent postclosure PAH (PP-PAH) was defined as a PASP that remained abnormal at 6 months postprocedure and persisted until the final follow-up (12). The Ethics Committee of the First Affiliated Hospital of Guangxi Medical University granted ethical approval (No. 2021; KY-E-156).

### Procedure

The procedure was performed under general anesthesia. The VSD and PDA were accessed via the right femoral vein and artery, and ASD was accessed via the right femoral vein only. Heparin (100 IU/kg) was given intravenously postfemoral cannulation. All patients underwent right and left cardiac catheterization for hemodynamic assessment. Blood gas analysis was conducted at each site. Qp/Qs and PVR were calculated using the estimated oxygen consumption. Angiography and intraoperative TTE were used to determine the location and size of the defect, which guided the occluder selection. In patients with a ratio of pulmonary arterial pressure to aortic pressure (Pp/Ps) > 0.8, we attempted transcatheter closure to assess reversibility. The following criteria were used (9): (1) a ≥ 20% reduction in mPAP or PASP; (2) stable aortic pressure; and (3) no worsening of symptoms such as dyspnea, irritability, pallor, chest pain, or heart rate drop. We monitored patients for at least 20 min during defect occlusion. The device was released if all criteria were met; otherwise, the occluder was retracted.

### Data collection

We gathered general information such as sex, age, body mass index, and main symptoms and signs from the medical records. Laboratory data, including levels of troponin I, electrolytes, cystatin, creatine kinase-MB, uric acid, and brain natriuretic peptide (BNP), were collected within 24 h following admission. Upon admission, each patient received TTE, and echocardiographic parameters, including the PASP, left atrial dimension (LAD), left ventricular end-diastolic dimension (LVEDD), pulmonary artery dimension (PAD), aortic dimension (AOD), left ventricular ejection fraction (LVEF), and right ventricular dimension (RVD), were documented. The x-ray data, including the cardiothoracic ratio and eminence of the PA segment, were also recorded. The hemodynamic data from cardiac catheterization before intervention were recorded.

### Follow-up

Patients received regular follow-up exams, including physical exams, electrocardiograms, x-rays, and TTE, at 24 h; 1, 3, 6, and 12 months; and then annually. The patients' records were thoroughly reviewed, and a phone survey was attempted for those lacking documented follow-up data.

### Statistical analysis

Data analysis was conducted using SPSS (version 26.0 for Windows, SPSS, Inc., Chicago, Illinois) and R (R version 4.2.1) software. The normality test was conducted using the Shapiro–Wilk test. Quantitative variables are shown as the mean ± standard deviation for normally distributed data or as the median with the interquartile range for skewed data. The Student's t test or analysis of variance was employed for quantitative variables with a normal distribution, whereas the Mann‒Whitney *U*-test or Kruskal‒Wallis H test was used otherwise. Categorical variables are presented as frequencies (percentages), and comparisons between groups were performed using Fisher's exact test or the chi-square test. Logistic regression analysis was conducted to assess the risk factors for PP-PAH. Factors with a *P* value less than 0.1 in the univariate analysis were incorporated into the multivariate logistic regression model. A *P* value less than 0.05 was regarded as statistically significant.

## Results

### Baseline characteristics

The study enrolled 210 children with CHD-PAH, including 87 males and 123 females. In children with PAH, the prevalence rates were 84.29% for mild cases (*n* = 177), 8.57% for moderate cases (*n* = 18), and 7.14% for severe cases (*n* = 15). Most moderate-to-severe PAH patients occurred in the PDA group, whereas all VSD patients had mild PAH. Ninety-two patients (43.81%) experienced recurrent respiratory infections, and eighty-two (39.05%) experienced growth retardation, with 28 (13.33%) experiencing shortness of breath and 21 experiencing fatigue (10.00%). Heart murmur was found in 97.14% of the patients, with 10.48% of the patients having an augmented P2. The patients had a mean mPAP of 30.05 ± 12.28 mmHg, a Pp/Ps ratio of 0.46 ± 0.19, and a median PVRI of 2.04 (1.44–3.21) wood units/m². While in the severe PAH group. Compared with those with mild PAH, patients with moderate and severe PAH presented an increased cardiothoracic ratio, LAD, LVEDD, PAD, and Pp/Ps (*P* < 0.05), but there were no significant differences across the groups in AOD, RVD, LVEF, mRAP, Qp/Qs, or PVRI. At baseline, 7 patients (3.33%) were receiving at least one pulmonary vasodilator, including 5 patients who were receiving bosentan and 2 who were receiving multiple agents (1 bosentan l + iloprost and 1 bosentan + sildenafil). Table 1 summarizes the baseline characteristics of the patients.

**Table 1:** Baseline characteristics of patients with CHD-PAH.

Parameters	All (*n* = 210)	Mild (*n* = 177)	Moderate (*n* = 18)	Severe (*n* = 15)	*P*
Ages (years)	4.08 (2.83–5.75)	4.25 (3.17–6.13)	1.33 (0.92–3.75)	2.83 (1.25–4.58)	**<0**.**001**
Male, *n* (%)	87 (41.43)	77 (43.50)	8 (44.44)	2 (13.33)	0.077
BMI (kg/m^2^)	15.02 ± 2.14	15.07 ± 2.04	15.00 ± 3.07	14.40 ± 1.86	0.487
Type of CHD, *n* (%)					**<0**.**001**
VSD	74 (35.24)	74 (41.81)	0 (0.00)	0 (0.00)	
ASD	65 (30.95)	64 (36.16)	1 (5.56)	0 (0.00)	
PDA	61 (29.05)	32 (18.08)	16 (88.89)	13 (86.67)	
Combination	10 (4.76)	7 (3.95)	1 (5.56)	2 (13.33)	
Symptoms and signs, *n* (%)					
Recurrent respiratory infections	92 (43.81)	67 (37.85)	6 (33.33%)	9 (60.00)	0.093
growth retardation	82 (39.05)	61 (34.46)	11 (61.11)	10 (66.67)	**0**.**011**
Shortness of breath	28 (13.33)	16 (9.04)	6 (33.33)	6 (40.00)	**<0**.**011**
Fatigue	21 (10.00)	18 (10.17)	1 (5.56)	2 (13.33)	0.714
Heart murmur	204 (97.14)	171 (96.61)	18 (100.00)	15 (100.00)	1.000
augmented P2	22 (10.48)	12 (6.78)	6 (33.33)	4 (26.67)	**0**.**001**
Cyanosis	29 (13.81)	18 (10.17)	5 (27.78)	6 (40.00)	**0**.**002**
x-ray					
Cardiothoracic ratio	0.56 ± 0.05	0.55 ± 0.05	0.61 ± 0.04*	0.62 ± 0.05*	**0**.**001**
Eminence of PA segment, *n* (%)	109 (51.90)	86 (48.59)	13 (72.22)	10 (66.67)	0.077
Echocardiography					
AOD (mm)	18.75 ± 2.92	18.82 ± 2.89	18.53 ± 3.04	18.33 ± 3.20	0.773
LAD (mm)	24.79 ± 5.18	24.16 ± 4.53	27.79 ± 7.11*^,#^	28.73 ± 7.09*^,#^	<**0**.**001**
LVEDD (mm)	38.33 ± 6.83	37.33 ± 6.11	42.74 ± 8.30*	44.53 ± 8.16*	**0**.**002**
RVD (mm)	17.69 ± 6.73	18.02 ± 7.12	15.84 ± 5.32	17.36 ± 5.34	0.414
PAD (mm)	21.25 ± 3.86	20.87 ± 3.63	22.84 ± 4.68*	23.20 ± 4.60*	**0**.**013**
LVEF (%)	73.40 ± 15.28	73.70 ± 16.37	72.26 ± 5.94	71.28 ± 6.64	0.794
PASP (mmHg)	40.06 ± 13.91	36.59 ± 8.55	51.89 ± 24.21*	60.57 ± 16.04*^,#^	**<0**.**001**
Hemodynamics					
PASP (mmHg)	43.83 ± 16.86	37.70 ± 8.78	68.79 ± 7.52*	84.20 ± 11.93*^,#^	**<0**.**001**
mPAP (mmHg)	30.05 ± 12.28	25.26 ± 4.51	48.53 ± 4.06*	62.87 ± 8.46*^,#^	**0**.**001**
Qp/Qs	2.18 (1.60–3.28)	2.18 (1.6–3.16)	1.73 (1.43–2.39)	3.78 (2.32–4.88)	0.058
PVRI (wood units/m^2^)	2.04 (1.44–3.21)	2.04 (1.46–3.15)	0.87 (1.91–3.57)	2.20 (1.32–4.78)	0.084
mRAP (mmHg)	7.18 (5.00–9.00)	7.05 (5.00–9.00)	7.50 (6.75–9.00)	7.00 (4.00–8.00)	0.240
Pp/Ps	0.46 ± 0.19	0.39 ± 0.11	0.77 ± 0.14*	0.85 ± 0.14*^,#^	**<0**.**001**
Drugs, *n* (%)					−
Bosentan	5 (2.38)	0 (0.00)	2 (11.11)	3 (20.00)	
Bosentan l + iloprost	1 (0.48)	0 (0.00)	0 (0.00)	1 (6.67)	
Bosentan + sildenafil	1 (0.48)	1 (0.56)	0 (0.00)	0 (0.00)	

Data are shown as mean ± standard deviation, median (interquartile range), or number (percentage), depending on the data type.

AOD, aortic dimension; ASD, atrial septal defect; BMI, body mass index; CHD-PAH, congenital heart disease complicated with pulmonary arterial hypertension; LAD, left atrial dimension; LVEDD, left ventricular end-diastolic dimension; LVEF, left ventricular ejection fraction; mPAP, mean pulmonary arterial pressure; mRAP, mean right atrial pressure; PAD, pulmonary artery dimension; PASP, pulmonary arterial systolic pressure; PDA, patent ductus arteriosus; Pp/Ps, ratio of pulmonary arterial pressure to aortic pressure; PVRI, pulmonary vascular resistance index; Qp/Qs, the ratio of pulmonary to the systemic circulation; RVD, right ventricular dimension; VSD, ventricular septal defect.

Bold values indicate statistically significant intergroup differences (*P* < 0.05).

^*^ *P* < 0.05 compared with mild group.

^#^ *P* < 0.05 compared with moderate group.

### Procedural outcomes

Among the 210 patients, 206 had successful interventions. The occlusion success rate could reach 98.10%. In one of the four cases in which closure failed, the patient was a 5.9-year-old girl with a 16 mm VSD. The procedure failed because of the large size of the VSD and insufficient aortic valve rim, and the patient was transferred for surgery repair. The second patient was a 4.6-year-old girl with an 11 mm VSD. The occluder was not well formed after being released. Finally, the intervention was stopped. The third patient was a 5.4-year-old boy with an 11 mm VSD. The device was malpositioned immediately after release, and a surgical emergency operation was performed to remove the occluder. During follow-up, the VSD tended to close, with a diameter of 2 mm at the last follow-up. The last patient was a 5.3-year-old girl with a 25 mm ASD who was treated with a 26 mm occluder. Third-degree atrioventricular block occurred during the closure process, and the plugging was suspended. After sinus rhythm was restored, a 24 mm occluder was selected for repeated occlusion, but it was not successful because the occluder was too small. Nine patients underwent trial occlusion, resulting in a decrease in mPAP from 55.44 to 31.89 mmHg and in PASP from 77.56 to 49.11 mmHg (*P* < 0.001), without affecting systemic blood pressure.

Early adverse events occurred in 12.14% (*n* = 25) of the patients. A residual shunt was detected in 2.91% (*n* = 6) of the patients, with most being small and temporary, persisting in only 2 patients. One patient required surgical repair at the 2-year follow-up due to an increased shunt volume, whereas another had a residual shunt at 18 months but showed no clinical symptoms. Arrhythmia occurred in 2.91% (*n* = 6) of the patients, including 2 left anterior branch blocks, 1 third-degree atrioventricular block (III°AVB), 1 II°AVB, 1 supraventricular tachycardia, and 1 atrial premature beat. Most patients normalized during follow-up, except for one patient who developed a complete left bundle branch block and II° AVB from a left anterior branch block. The patient experienced syncope once during follow-up, and since the parent declined pacemaker treatment, the occluder was removed and surgical repair performed. One patient experienced a temporary drop in heart rate and blood oxygen during the procedure, which improved after atropine administration. One patient experienced bleeding and required a blood transfusion. Other complications included 4 cases of stress fever, 4 side effects from intravenous anesthesia, 1 case of hemolysis, 1 case of myocardial injury, and 1 case of femoral fistula, all of which improved before discharge. A summary is provided in Table 2.

**Table 2:** Early complications and their distributions.

Complications	No (%)
Arrhythmia	6 (2.91)
Left anterior branch block	2 (0.97)
Third-degree atrioventricular block	1 (0.49)
Second-degree atrioventricular block	1 (0.49)
Supraventricular tachycardia	1 (0.49)
Atrial premature beat	1 (0.49)
Residual shunt	6 (2.91)
Stress fever	4 (1.94)
Side effects of intravenous anesthesia	4 (1.94)
Hemolysis	1 (0.49)
Myocardial injury	1 (0.49)
Femoral fistula	1 (0.49)
Heart rate and blood oxygen decreases	1 (0.49)
Blood transfusion	1 (0.49)

### Follow-Up

After the intervention, all 25 patients received pulmonary vasodilators: 15 were on monotherapy (13 with bosentan, 2 with beraprost sodium) and 10 were on combination therapy (8 with bosentan + beraprost sodium, 1 with bosentan + iloprost, 1 with bosentan + sildenafil). During a follow-up of 1.25 to 10.50 years, no deaths occurred, and the occluding devices remained stable and properly shaped. No cases of infective endocarditis, heart failure, thromboembolism, new arrhythmias, or device-related atrioventricular valve regurgitation were reported.

**Figure 1:**
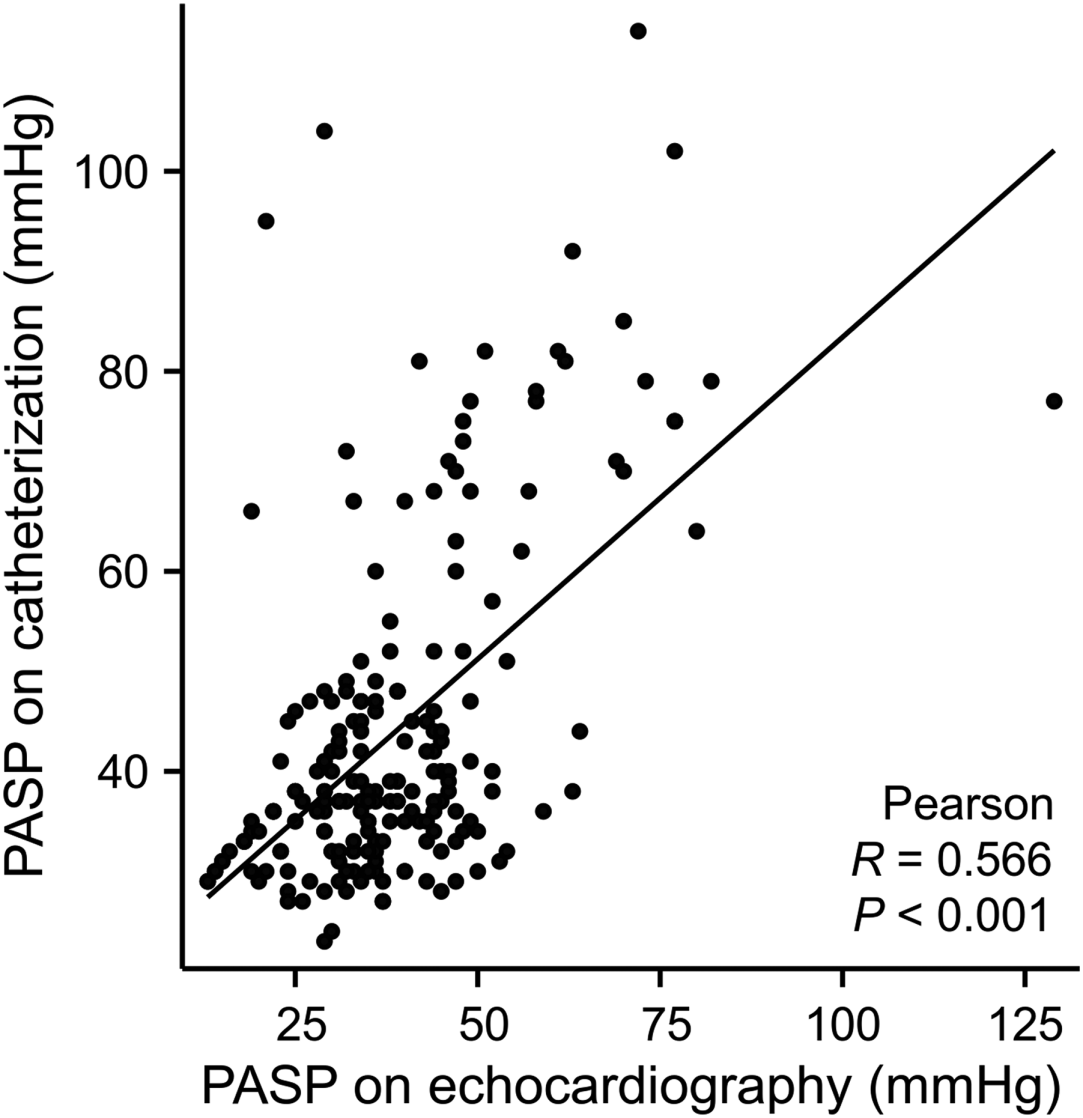
Correlation analysis between pulmonary arterial systolic pressure measured by echocardiography vs. cardiac catheterization.

**Figure 2:**
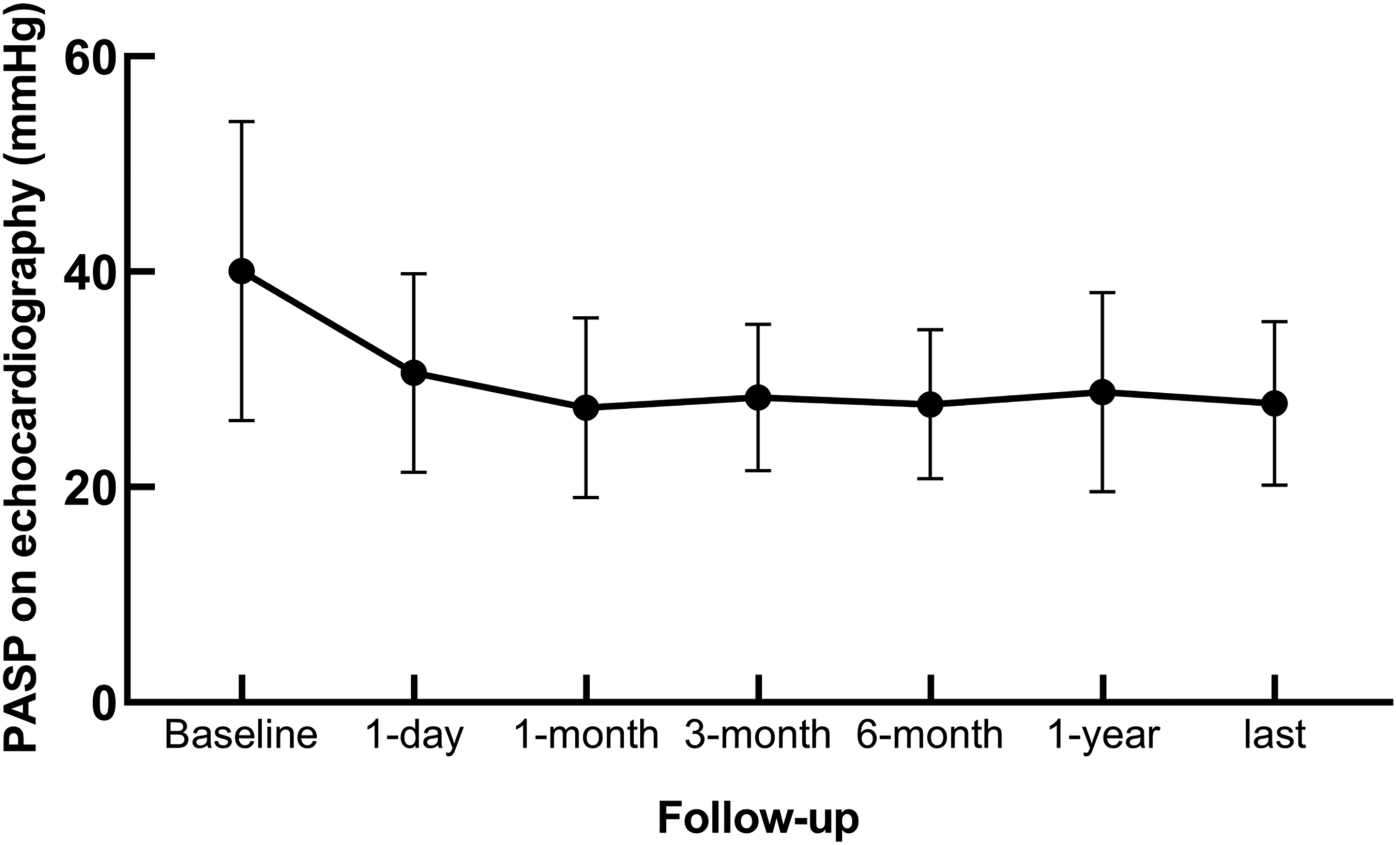
Changes in pulmonary arterial systolic pressure in patients with congenital heart disease complicated with pulmonary arterial hypertension.

**Figure 3:**
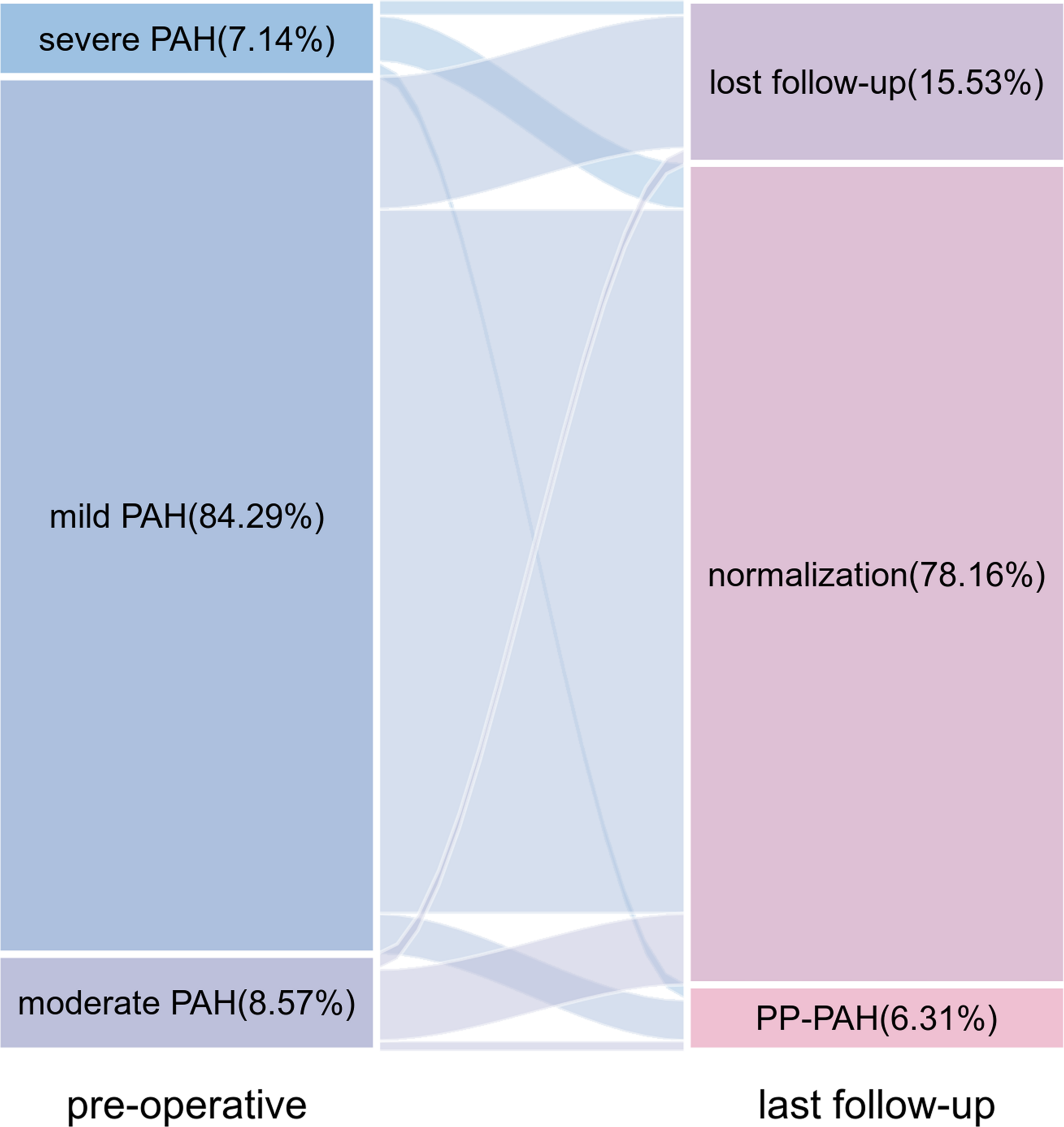
Sankey diagram of changes in pulmonary arterial hypertension from baseline to the last follow-up.

PAH was monitored by TTE postoperatively. Correlation analysis revealed a positive relationship between PASP measurements obtained via TTE and heart catheterization (*r* = 0.566, *P* < 0.001; Figure 1). The PAP decreased significantly after the intervention and stabilized over time (Figure 2). Thirty-four patients had PAH 24 h postintervention; 15 remained affected at 3 months, and 13 remained affected at the final follow-up. Out of 206 patients who underwent transcatheter closure, 13 developed PP-PAH, 32 were lost to follow-up, and 161 had normal pulmonary pressure. Among 15 with severe PAH, 3 developed PP-PAH, 3 were lost to follow-up, and the rest normalized. Figure 3 illustrates the trends in PAH before and after the intervention. Significant decreases in LAD, RVD, PAD, LVEF, PASP, and the cardiothoracic ratio were observed at the 1-month, 3-month, 6-month, 1-year, and 2-year follow-ups (*P* < 0.05, Table 3). The AOD and LVEDD significantly changed at 1 year but not at 2 years (*P* > 0.05).

**Table 3:** Echocardiography and x-ray data during follow-up.

Parameters	Preoperative	Postoperative 1 m	Postoperative 3 m	Postoperative 6 m	Postoperative 1 y	Postoperative 2 y
CTR	0.56 ± 0.05	0.54 ± 0.04*	0.54 ± 0.04*	0.54 ± 0.03*	0.53 ± 0.04*	0.53 ± 0.05*
AOD	18.75 ± 2.92	17.41 ± 3.05*	17.78 ± 2.97*	17.51 ± 3.22*	17.80 ± 3.17*	18.86 ± 2.79
LAD	24.79 ± 5.18	21.35 ± 4.22*	22.00 ± 4.02*	21.44 ± 3.94*	21.37 ± 3.19*	22.39 ± 4.17*
LVEDD	38.33 ± 6.83	35.08 ± 5.11*	35.07 ± 6.23*	35.12 ± 4.94*	35.12 ± 4.94*	37.14 ± 4.33
RVD	17.69 ± 6.73	14.41 ± 3.53*	14.43 ± 3.33*	14.15 ± 2.68*	14.15 ± 2.68*	15.20 ± 3.22*
PAD	21.25 ± 3.86	17.20 ± 3.06*	17.34 ± 2.93*	16.99 ± 3.04*	17.76 ± 2.98*	17.98 ± 2.81*
LVEF	73.40 ± 15.28	68.49 ± 6.91*	69.67 ± 5.64*	69.47 ± 7.26*	69.96 ± 7.59*	68.78 ± 5.03*
PASP	40.06 ± 13.91	27.36 ± 8.35*	28.33 ± 6.80*	27.68 ± 6.92*	28.81 ± 9.25*	28.20 ± 7.03*

The data are presented as the mean ± standard deviation.

AOD, aortic dimension; CTR, cardiothoracic ratio; LAD, left atrial dimension; LVEDD, left ventricular end-diastolic dimension; LVEF, left ventricular ejection fraction; PAD, pulmonary artery dimension; PASP, pulmonary arterial systolic pressure; RVD, right ventricular dimension.

* *P* < 0.05.

### Factors associated with PP-PAH

**Figure 4:**
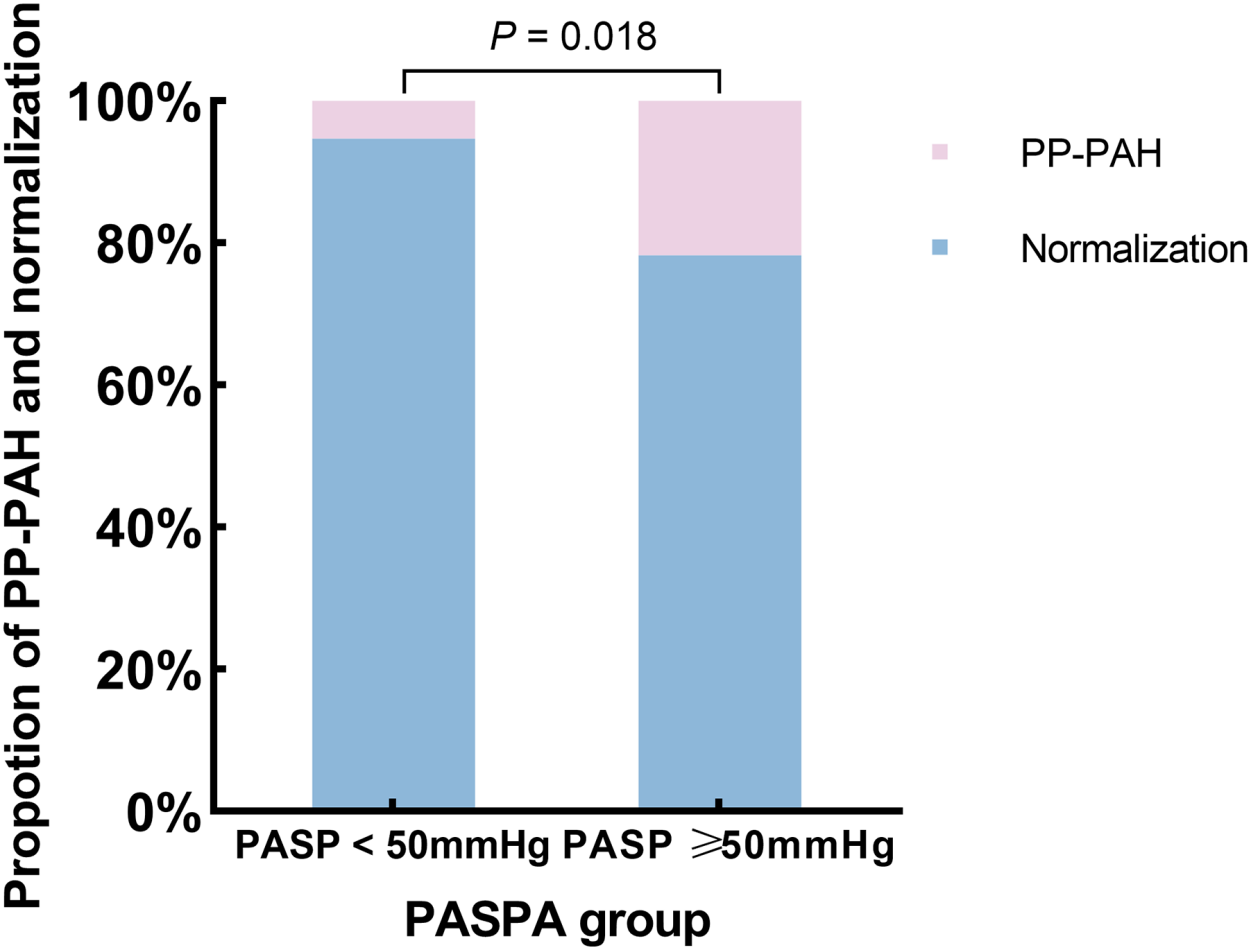
Proportion of PP-PAH in different PASP group.

Univariate analysis indicated that RVD was linked to PP-PAH [odds ratio [OR] = 1.096, 95% confidence interval [CI] = 1.029–1.168, *P* = 0.004]. Stepwise selection was used for further analysis. Multivariate analysis revealed that an increase in the PASP of 1 mmHg increased the risk of PP-PAH by 3.3% (OR = 1.033, 95% CI = 1.005–1.061; *P* = 0.019). Additionally, for each 1 millimeter increase in RVD, the risk of PP-PAH increased by 1.1-fold (OR = 1.111, 95% CI = 1.039–1.187, *P* = 0.002) (Table 4). To assess the probability of PP-PAH across different baseline PASP levels, patients were stratified into two groups: PASP ≥ 50 mmHg and PASP < 50 mmHg. The incidence of PP-PAH was significantly higher in the PASP ≥ 50 mmHg group compared to the PASP < 50 mmHg group (21.74% vs. 5.30%, *P* < 0.05, Figure 4).

**Table 4:** Logistic regression analysis on risk factors of PP-PAH.

Characteristics	Univariate Cox regression analysis			Multivariate Cox regression analysis		
	OR	95% CI	*P*	OR	95% CI	*P*
Age, per year	1.076	0.913–1.267	0.383			
Female (reference: male)	1.211	0.392–3.738	0.253			
BMI	1.061	0.836–1.347	0.626			
AOD	0.993	0.818–1.206	0.946			
PAD	1.042	0.905–1.200	0.566			
RVD	1.096	1.029–1.168	**0**.**004**	1.111	1.039–1.187	**0**.**002**
PASP	1.026	1.000–1.053	0.052	1.033	1.005–1.061	**0**.**019**
Qp/Qs	1.116	0.957–1.302	0.160			
Pp/Ps	3.570	0.225–56.614	0.367			
mPAP	1.031	0.995–1.068	0.093			
PVRI	0.902	0.564–1.443	0.123			
Cystain C	0.808	0.300–22.018	0.899			
Uric acid	1.001	0.993–1.008	0.878			

AOD, aortic dimension; BMI, body mass index; CI, confidence interval; mPAP, mean pulmonary arterial pressure; OR, odds ratio; PAD, pulmonary artery dimension; PASP, pulmonary arterial systolic pressure; PP-PAH, persistent postclosure PAH; Pp/Ps, ratio of pulmonary artery pressure to aortic pressure; PVRI, pulmonary vascular resistance index; Qp/Qs, the ratio of pulmonary to the systemic circulation; RVD, right ventricular dimension.

Bold values indicate statistically significant intergroup differences (*P* < 0.05).

## Discussion

In the present study, the clinical characteristics and outcomes of children with CHD-PAH who underwent percutaneous defect closure were assessed at a tertiary medical center in Guangxi, China. The main finding was that transcatheter closure was effective and yielded positive results in selected patients with CHD-PAH. Although PASP generally improved after closure, some patients still experienced PAH after defect correction, which was linked to the baseline PASP and RVD.

Transcatheter closure for CHD has been shown to be an effective therapy for the wide spectrum of CHD, with advancements in techniques leading to a high success rate. This study revealed a 98.10% success rate for interventional blockade in CHD-PAH patients, which is consistent with the literature (11, 19–22). At 24 h postprocedure, PAH had completely regressed in 83.50% of patients. Over time, PAP gradually normalized; however, approximately 6.31% of the patients still presented with PAH at the last follow-up, which was regarded as PP-PAH. Comparative studies related to this topic are limited. Several studies have reported an estimated prevalence of PAH postclosure ranging from 13%–25%, which is higher than that obtained in our study (8, 12, 23). This discrepancy may be attributed to variations in the inclusion criteria. In our study, the majority of participants presented with mild PAH, whereas other studies primarily included individuals with moderate to severe PAH. Additionally, the number of heart chambers and the diameters of the pulmonary artery and aorta significantly decreased and normalized, which is consistent with previous studies (11, 24). Therefore, transcatheter closure of CHD-PAH has a definite effect.

The presence of a residual shunt is the key indicator for evaluating the success of transcatheter closure in CHD patients. This study revealed that the incidence of immediate residual shunt was 2.91%, which is lower than previously reported rates (11, 20, 25, 26). Most shunts were small and resolved in 4 patients within 1 year, although 1 patient had a persistent shunt at 18 months without symptoms, and another patient required surgery at 2 years owing to an increased shunt volume. Residual shunts often result from incorrect occluder sizing, so selecting the appropriate occluder type and location is essential to ensure complete closure without impacting aortic and atrioventricular valve functions.

Arrhythmia, which affected 2.91% of the patients in the present study, is a common complication of transcatheter closure, which is consistent with previous cardiac catheterization studies in CHD (27). There were two cases of left anterior branch block, one case each of third-degree and second-degree atrioventricular block, one case of supraventricular tachycardia, and one atrial premature beat. Most patients normalized during follow-up, except for one patient who progressed to complete left bundle branch block and II° AVB from a left anterior branch block. The issue was resolved after removing the occluder and performing surgical repair. Postoperative arrhythmia may result from temporary inflammation or edema at the device site, direct compression trauma, or scar formation in the conduction tissue (28). Steroid administration has been shown to have a positive effect on postoperative arrhythmias (20, 28). To prevent arrhythmia, prolonged intracardiac procedures should be avoided, and an occluder that is slightly larger (by 1–2 mm) than the defect should be used to prevent tissue compression and edema.

Complications resulting from femoral artery access in cardiac catheterization may include bleeding, hematoma, and arteriovenous fistula. In this study, one patient developed an arteriovenous fistula, and another needed blood transfusion due to bleeding. Other adverse events included stress fever, anesthesia side effects, hemolysis, myocardial injury, and temporary heart rate drops, all of which improved before discharge. Postclosure, no infective endocarditis, heart failure, thromboembolism, occluder issues, or deaths occurred during follow-up. Transcatheter closure for CHD-PAH has few complications and favorable safety.

PAH is a common complication of CHD, particularly in patients with uncorrected systemic-to-pulmonary shunts (29). Increased pulmonary blood flow is considered a crucial trigger for pulmonary vascular remodeling, leading to irreversible changes in the pulmonary vasculature (30). Timely correction of the shunt may lead to full disease regression, but this potential is lost after a certain point (3). Closure beyond the reversible phase may increase the mortality risk and worsen the prognosis compared with patients with uncorrected CHD-PAH (5). Approximately 10% of children with CHD and PAH are considered ineligible for shunt closure (3). These findings highlight the importance of differentiating reversible from irreversible PAH. Currently, Qp/Qs < 1.5 and PVRI > 8 wood units/m^2^ are regarded as contraindications for shunt closure (6, 7). However, these values were derived using the oximetric shunt formula, which relies on measurements from collected arterial and venous blood gases and is susceptible to calculation errors. The current guidelines recommend the use of a multiparametric approach that considers all the available information, rather than relying solely on hemodynamic data, in decisions regarding shunt closure (31). In this study, none of the patients had cyanosis or symptoms related to heart failure. Echocardiography revealed a left-to-right shunt in blood flow. The median PVRI for all patients was 2.04 wood units/m^2^, and 2.20 wood units/m^2^ in the severe PAH subgroup. Notably, all patients had a PVRI below 6 wood units/m^2^, aligning with international guidelines for defect closure. Among 33 patients with moderate-to-severe PAH, 9 underwent trial occlusion, achieving a ≥20% reduction in mPAP and/or PASP without affecting systemic blood pressure, thus meeting the closure criteria. Although the patients were assessed as correctable in the present study; 13 still had persistent PAH after closure. PAH can occur much later than 1 to 10 years after intervention (5). A prior study revealed that the cumulative incidence of PH after closure increased over time even in patients with mild defects (32). These alarming results support the monitoring of all CHD-PAH patients after shunt closure, even those with mild PAH.

This study revealed that preoperative PASP and RVD are risk factors for PP-PAH. Sadiq et al. (19) reported that a PASP with oxygen ≤75 mmHg is associated with the regression of PAH. The substantial increase in PAP before intervention indicates that the recovery from PAH-related vascular changes is slow, even after shunt interruption. Some patients may also experience left heart insufficiency before or after surgery due to the increased load. Consequently, it takes a considerable amount of time for PAP to normalize postsurgery. To the best of our knowledge, this is the first study to identify RVD as a risk factor for persistent PAH after intervention. Previous studies have shown that RVD is associated with adverse clinical outcomes (33, 34). RV pressure overload leads to RV dilation, impairing RV function and increasing tricuspid valve regurgitation, which in turn worsens RV dilation (33). These findings necessitate additional research with larger sample sizes and extended follow-up due to the relatively small PP-PAH sample size.

Target therapies had shown favorable functional and haemodynamic results in patients with PAH (31). Compared with other group 1 PAH subgroups, there is limited evidence on the use of PAH-approved medications in patients with CHD-PAH. Experts recommended PAH therapies for: (1) lowering PVR for safe repair, (2) post-repair treatment, (3) improving quality of life in patients with Eisenmenger syndrome, and (4) reducing PAP and PVR in patients with pulmonary hypertensive vascular disease (35). Currently, evidence-based treatment data for PAH with systemic-to-pumonary remain relatively limited. Treatment decisions often rely more on expert clinical experience. In this study, 25 patients with moderate to severe PAH were treated with pulmonary vasodilators. Seven were treated before intervention due to a PASP over 70 mmHg measured by TTE, while 18 received therapy post-intervention based on mPAP from cardiac catheterization. At the last follow-up, 4 out of 25 patients still had PAH, while the rest returned to normal. No drug-related adverse effects were noted. Rong et al. (11) suggested that patients with moderate to severe PAH require early targeted drug interventions post-surgery. However, the impact of PAH therapies on patients with systemic-to-pulmonary shunts is not well understood. More extensive multicenter studies are needed to confirm the efficacy and safety of these treatments for this group.

Heart catheterization is the gold standard for PAH diagnosis, but its invasive nature makes it unsuitable for repeated assessments (36). Only a few patients receive repeated cardiac catheterization after the procedure (11, 12). The TTE estimates PASP by measuring peak tricuspid regurgitation velocity, offering an alternative for assessing PAH after closure. Multiple studies have demonstrated good consistency between PASP estimated by TEE and values obtained through cardiac catheterization (9, 11, 37). In our study, the PASP estimated by TTE correlated well with cardiac catheterization measurements, indicating that TTE was a reliable method for assessing PAH postintervention.

## Limitations

There were several limitations in this study. First, selection bias was inevitable because the study was retrospective. Second, the PP-PAH sample size was relatively small. Therefore, the findings should be considered preliminary. Larger samples and extended follow-up are necessary for further validation of these findings. Third, TTE assessment rather than catheterization was selected to measure the PASP after intervention.

## Conclusions

This study revealed that transcatheter closure is an effective and safe therapeutic option for children with correctable CHD-PAH. Preoperative PASP and RVD are risk factors for PP-PAH. Long-term follow-up is essential for all CHD-PAH patients after shunt closure.

## Data Availability

The raw data supporting the conclusions of this article will be made available by the authors, without undue reservation.
